# Glyoxylate cycle gene *ICL1* is essential for the metabolic flexibility and virulence of *Candida glabrata*

**DOI:** 10.1038/s41598-019-39117-1

**Published:** 2019-02-26

**Authors:** Shu Yih Chew, Kok Lian Ho, Yoke Kqueen Cheah, Tzu Shan Ng, Doblin Sandai, Alistair J. P. Brown, Leslie Thian Lung Than

**Affiliations:** 1 Department of Medical Microbiology and Parasitology, Faculty of Medicine and Health Sciences, Universiti Putra Malaysia, 43400 UPM Serdang, Selangor, Malaysia; 2 Department of Pathology, Faculty of Medicine and Health Sciences, Universiti Putra Malaysia, 43400 UPM Serdang, Selangor Malaysia; 3 Department of Biomedical Sciences, Faculty of Medicine and Health Sciences, Universiti Putra Malaysia, 43400 UPM Serdang, Selangor Malaysia; 4 Department of Molecular Medicine, Faculty of Medicine, University of Malaya, 50603 Kuala Lumpur, Wilayah Persekutuan, Kuala Lumpur, Malaysia; 5 Infectomics Cluster, Advanced Medical and Dental Institute, Universiti Sains Malaysia, 13200 Kepala Batas, Pulau Pinang Malaysia; 6 MRC Centre for Medical Mycology at the University of Aberdeen, Institute of Medical Sciences, Foresterhill, Aberdeen AB25 2ZD United Kingdom

## Abstract

The human fungal pathogen *Candida glabrata* appears to utilise unique stealth, evasion and persistence strategies in subverting the onslaught of host immune response during systemic infection. However, macrophages actively deprive the intracellular fungal pathogen of glucose, and therefore alternative carbon sources probably support the growth and survival of engulfed *C. glabrata*. The present study aimed to investigate the role of the glyoxylate cycle gene *ICL1* in alternative carbon utilisation and its importance for the virulence of *C. glabrata*. The data showed that disruption of *ICL1* rendered *C. glabrata* unable to utilise acetate, ethanol or oleic acid. In addition, *C. glabrata icl1*∆ cells displayed significantly reduced biofilm growth in the presence of several alternative carbon sources. It was also found that *ICL1* is crucial for the survival of *C. glabrata* in response to macrophage engulfment. Disruption of *ICL1* also conferred a severe attenuation in the virulence of *C. glabrata* in the mouse model of invasive candidiasis. In conclusion, a functional glyoxylate cycle is essential for *C. glabrata* to utilise certain alternative carbon sources *in vitro* and to display full virulence *in vivo*. This reinforces the view that antifungal drugs that target fungal *Icl1* have potential for future therapeutic intervention.

## Introduction

Invasive candidiasis is a potentially lethal fungal infection caused by fungi from the *Candida* genus that is associated with high morbidity and mortality. Life-threatening blood stream infections (candidaemia) and deep-seated candidiasis are commonly seen in critically ill individuals such as intensive care unit (ICU) patients with predisposing host factors or underlying malignant diseases^1,2^. Over the last decade, the proportion of invasive candidiases caused by the predominant species *Candida albicans* has decreased. Meanwhile, there has been a corresponding shift towards certain non-*Candida albicans Candida* (NCAC) species, probably due to the selection imposed by antifungal drugs^2–4^. *Candida glabrata* has emerged as one of the most prominent invasive candidiasis-causing species, particularly in some of the European countries, USA, Canada and Australia^5^.

Numerous studies have focussed on *C. albicans*, and less attention has been devoted to the pathogenic attributes of *C. glabrata*. In *C. albicans*, hypha-mediated penetration is crucial for the invasion of the host epithelial cells through protruding filaments and secretion of hydrolytic enzymes and candidalysin^6,7^. In addition, the aggressive nature of *C. albicans* leads to stronger pro-inflammatory cytokine responses in the host. In contrast, *C, glabrata* is a haploid, non-dimorphic fungus that is incapable of hypha formation, and this pathogen seems to favour a ‘stealth and concealment’ approach during infection to avoid direct confrontation with immune cells^8^. Despite the lower pathogenicity of *C. glabrata* in comparison to *C. albicans*, the high mortality rate associated with invasive candidiasis caused by *C. glabrata* would argue otherwise. Therefore, *C. glabrata* likely possesses potent pathogenic attributes that do not relate to phenotypic dimorphism.

Interestingly, *C. glabrata* has been shown to elicit a unique cytokine profile that promotes the recruitment of monocytes instead of neutrophils^9^. Since *C. glabrata* survives and replicates within the hostile microenvironment of macrophages, but not in neutrophils^10,11^, it is possible that *C. glabrata* exploits these immune cells to survive against the neutrophil onslaught during the establishment of an infection. Upon engulfment by macrophages, *C. glabrata* reprograms its metabolic activity in order to adapt to nutrient deprivation (e.g. carbon starvation). Roetzer *et al*.^12^ have reported that *C. glabrata* counteracts nutrient deprivation via mobilization of intracellular resources through autophagy^12^. Autophagy, particularly pexophagy, is an important virulence factor in *C. glabrata* that is crucial to sustain this pathogen during carbon starvation. In addition, Ng *et al*.^13^ have shown that *SNF3*, which encodes a high affinity glucose sensor, is also important for *C. glabrata* to thrive within macrophages - a microenvironment with limited glucose availability^13^. In addition to autophagy and enhanced glucose sensing, alternative carbon utilisation is believed to be important for the survival and pathogenicity of *Candida* species. Transcriptional analyses of *C*. *albicans* and *C. glabrata* revealed extensive metabolic reprogramming that reflects adaptation to nutrient deprivation following macrophages engulfment^14,15^. This reprogramming includes the upregulation of genes from three interconnected alternative carbon utilisation pathways: gluconeogenesis (*FBP1* and *PCK1*), the glyoxylate cycle (*ICL1* and *MLS1*) and fatty acid β-oxidation (*FOX2* and *POX1*). Upregulation of these pathways indicates that the macrophage actively deprives *C. albicans* and *C. glabrata* of their preferable carbon source, thus forcing these fungal pathogens to tune their metabolism to alternative carbon sources.

The ability to utilise alternative carbon sources is important for *C. glabrata* in many host niches. For example, lactate assimilation is required for the survival in the intestine^16^. In addition, it has been shown that vaginal isolates of *C. glabrata* are able to utilise acetate, even in the presence of glucose^17^. The scavenging of alternative carbon sources, such as acetate, is dependent on a functional glyoxylate cycle^18^. The glyoxylate cycle bypasses the two decarboxylation steps in the tricarboxylic (TCA) cycle, thereby permitting the assimilation of this carbon. The glyoxylate cycle depends upon two enzymes, isocitrate lyase and malate synthase, to produce malate, an intermediate of the TCA cycle. The glyoxylate cycle is absent from mammalian tissues, but is conserved in protists, archaea, plants, bacteria, fungi and nematodes^19^. Muñoz-Elías & McKinney (2005) showed that disruption of the genes that encode ICL isoforms in bacterial pathogens, *ICL1* and *ICL2*, impairs the growth and persistence of *Mycobacterium tuberculosis in vivo*^20^. Mutants lacking both *icl1* and *icl2* showed a defect in intracellular replication and were rapidly eliminated from the mice. Isocitrate lyase is also required for *Salmonella enterica* serovar Typhimurium during chronic infection and is essential for the virulence of *Rhodococcus equi* and *Pseudomonas aeruginosa*^21–23^.

With regard to medically important fungi, isocitrate lyase is required for the growth of *Aspergillus fumigatus* on alternative carbon sources such as acetate, ethanol and fatty acids^24^. Nevertheless, isocitrate lyase is not required for the establishment of invasive aspergillosis in murine model^25^. Similarly, although *ICL1* is highly induced in the presence of alternative carbon sources and in rabbit meningitis model, *ICL1* mutants of *Cryptococcus neoformans* show no apparent virulence defect in murine or rabbit infection models *in vivo*^26^. In *C. albicans*, disruption of the key glyoxylate cycle gene *ICL1* severely attenuates virulence in murine models of invasive candidiasis^27,28^. These studies suggest that the degree to which the glyoxylate cycle contributes to human pathogenicity depends on the species of fungal pathogen. To date, the significance of the glyoxylate cycle in *C. glabrata* pathogenicity remains unknown. Therefore, taking cues from *C. albicans*, we have investigated the role of *ICL1* in the metabolic flexibility and virulence of *C. glabrata*.

## Results

### *ICL1* is essential for the growth of *C. glabrata* on certain alternative carbon sources

**Figure 1:**
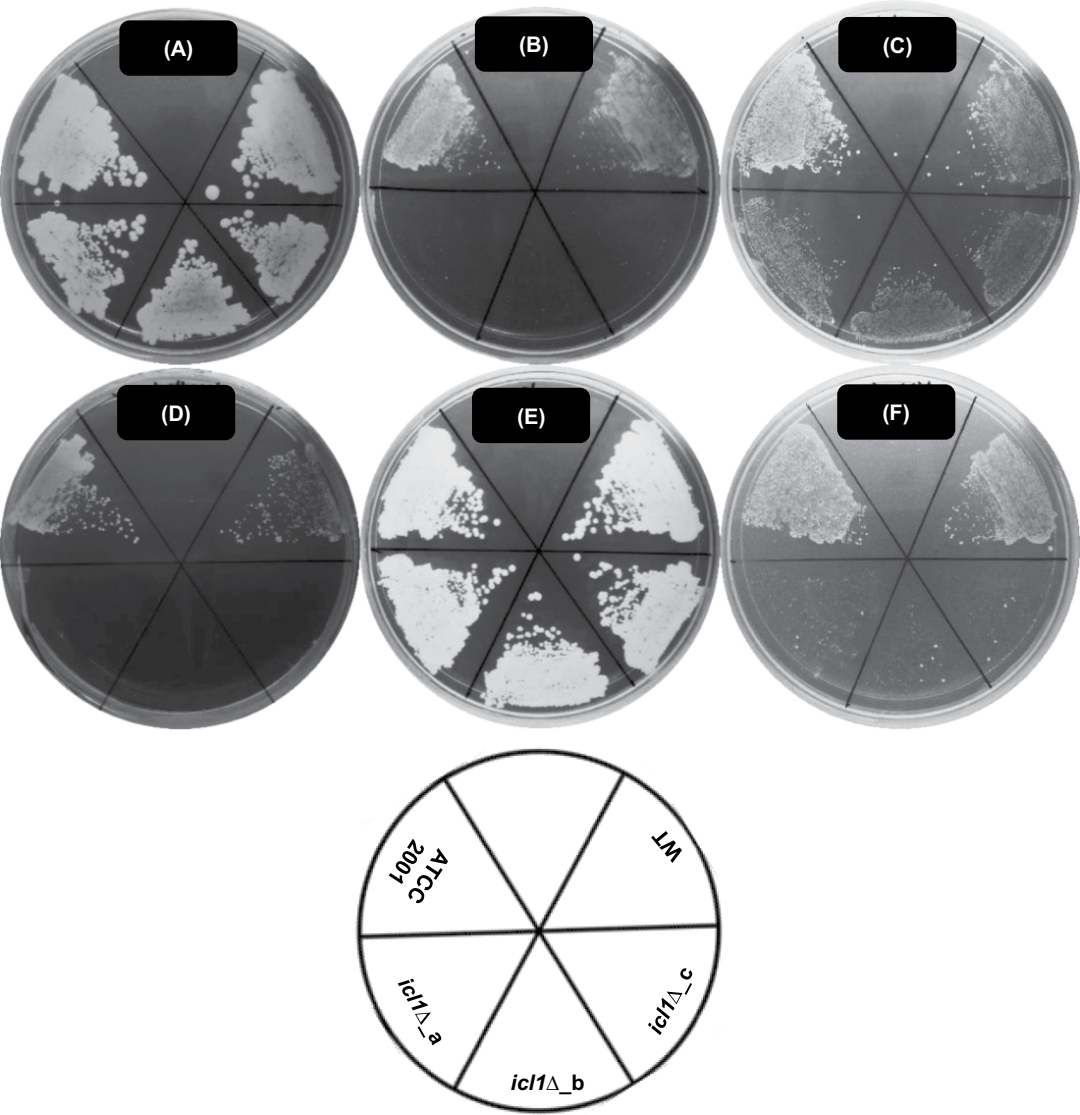
Representative image of growth phenotypes of *C. glabrata* ATCC 2001, WT and *icl1*∆ mutants (*icl1*∆_a, *icl1*∆_b and *icl1*∆_c) on glucose and alternative carbon sources. *C. glabrata* strains were grown on SC media containing (**A**) 2% glucose, (**B**) 2% acetate, (**C**) 2% lactate, (**D**) 2% ethanol, (**E**) 2% glycerol or (**F**) 0.2% oleic acid as the sole carbon source for 96 h at 37 °C. All experiments were performed in triplicate and each independent experiment was repeated three times.

**Figure 2:**
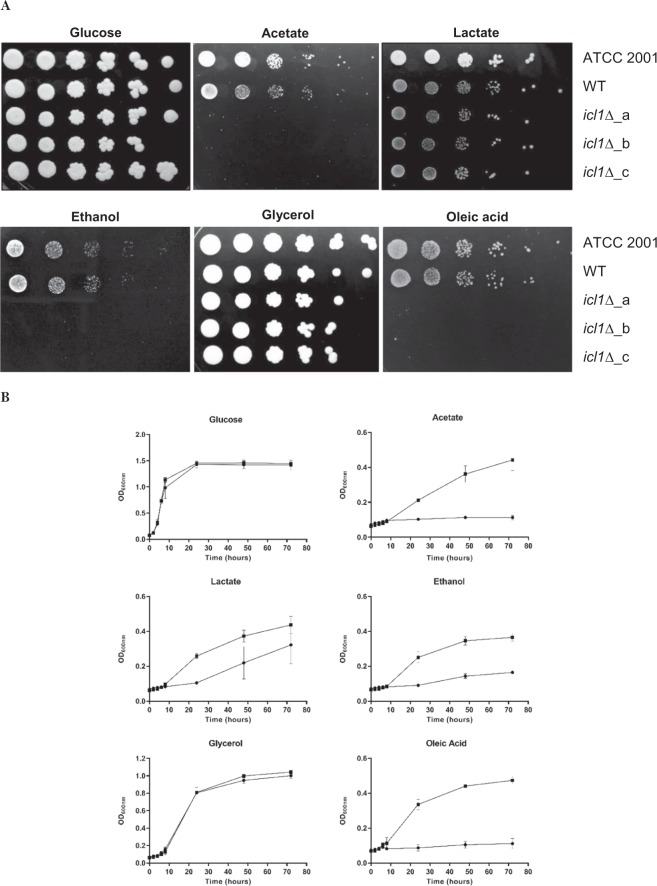
(**A**) Growth of *C. glabrata* ATCC 2001, WT and *icl1*∆ mutants in spot dilution assay. Deletion of *ICL1* renders *C. glabrata* unable to utilise and grow in SC media supplemented with 2% acetate, 2% ethanol or 0.2% oleic acids as the sole carbon source. (**B**) Growth profile of *C. glabrata* WT (■) and *icl1*∆ mutants (●) in liquid SC media supplemented with 2% glucose, 2% acetate, 2% lactate, 2% ethanol, 2% glycerol or 0.2% oleic acid as the sole carbon source. All experiments were performed in triplicate and each independent experiment was repeated three times.

First, the ability of *C. glabrata* ATCC 2001, WT and *icl1∆* cells to grow on glucose or alternative carbon sources was tested using simple growth assays. As anticipated, *C. glabrata* ATCC 2001 is able to utilise all of the alternative carbon sources tested, in addition to the preferred carbon source, glucose. The WT and mutant strains lacking *ICL1* were all viable and they grew equally well in the presence of glucose or glycerol as sole carbon source. There was a slight decrease in the growth of mutant strains on lactate (Figs 1 and 2). We also found that *ICL1* deletion rendered *C. glabrata* unable to grow on acetate and ethanol as sole carbon source (Figs 1 and 2). In addition, *C. glabrata icl1∆* cells grew poorly in media containing oleic acid (Figs 1 and 2). Similar carbon utilisation profiles were obtained for three independently constructed *C. glabrata icl1* mutants (*icl1∆_*a, *icl1∆_*b and *icl1∆_*c). We conclude that, in *C. glabrata*, *ICL1* is indispensable for the utilisation of acetate, ethanol and oleic acid, and partially required for the utilisation of lactate.

### *ICL1* is essential for the formation of *C. glabrata* biofilms in certain alternative carbon sources

**Figure 3:**
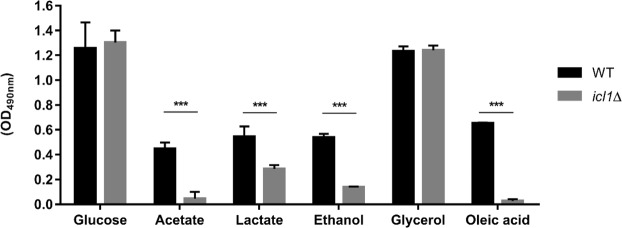
Biofilm formation of *C. glabrata* WT and *icl1*∆ mutants in glucose and alternative carbon sources. Results are presented as means ± SD. *p < 0.05 was considered statistically significant relative to WT strain. All experiments were performed in triplicate and each independent experiment was repeated three times.

Since the deletion of *ICL1* impacts the planktonic growth of *C. glabrata* on several alternative carbon sources, we then investigated the role of *ICL1* in biofilm formation. To achieve this, we measured the metabolic activity of *C. glabrata* biofilms formed on different alternative carbon sources. As expected, *icl1*∆ cells displayed similar levels of biofilm formation to the WT control strain during growth on glucose (Fig. 3). In addition, *ICL1* was not essential for biofilm formation in the presence of glycerol as the sole carbon source (Fig. 3). However, the disruption of *ICL1* reduced *C. glabrata* biofilm formation on acetate, lactate, ethanol and oleic acid. Indeed, significant reductions in biofilm formation were observed for the *icl1*∆ cells on oleic acid (up to 95%: *p* < 0.001), closely followed by acetate (90%), ethanol (75%) and lactate (48%).

### *ICL1* is essential for the survival of *C. glabrata* cells following macrophage engulfment

**Figure 4:**
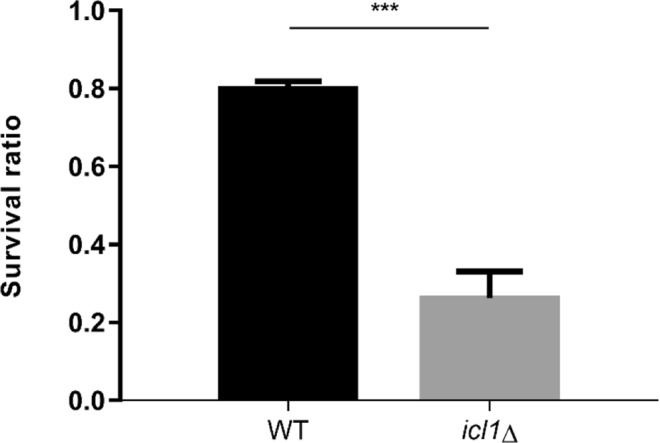
Survival ratio of internalised *C. glabrata* WT and *icl1*∆ mutants recovered from RAW264.7 macrophages. Results are presented as means ± SD. *p < 0.05 was considered statistically significant relative to WT strain. All experiments were performed in triplicate and each independent experiment was repeated three times.

Our results demonstrate that *ICL1* is required for the metabolic flexibility of *C. glabrata*. Therefore, we reasoned that *ICL1* might also play an essential role in promoting the survival of this fungus following phagocytosis by macrophages. RAW264.7 macrophages were challenged with *C. glabrata* and the survival of internalized fungal cells was determined by measuring the resultant colony forming units (CFUs). The results showed the *icl1*∆ mutant was much more susceptible to macrophage killing than the WT control strain (Fig. 4). This observation confirms the importance of *ICL1* for the survival of *C. glabrata* following macrophage ingestion.

### *ICL1* is essential for the virulence of *C. glabrata in vivo*

**Figure 5:**
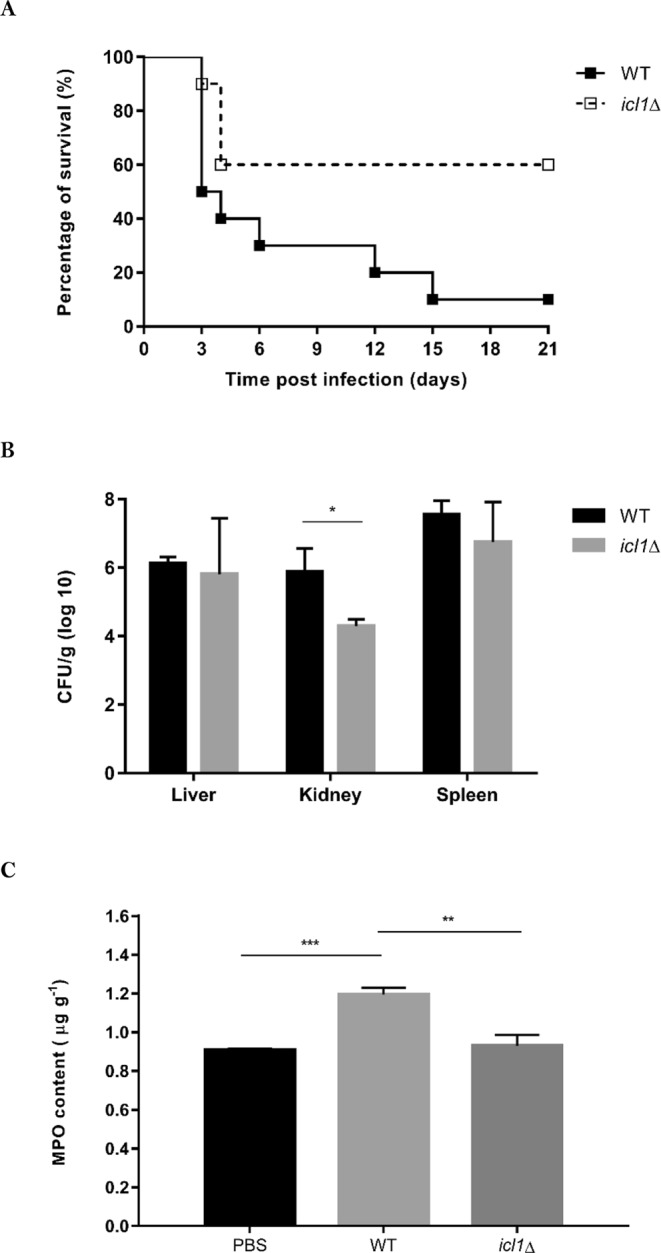
*ICL1* is essential to maintain wild type level of *C. glabrata* virulence *in vivo*. (**A**) Survival curve of immunosuppressed ICR mice infected with *C. glabrata* WT (n = 10) or *icl1*∆ mutant (n = 10). Mantel-Cox log rank analysis of survival curve demonstrated that the virulence of *icl1*∆ mutant was significantly attenuated (*p* < 0.05) compared to WT strain. (**B**) Fungal burdens in different organs harvested from immunosuppressed ICR mice infected with *C. glabrata* WT or *icl1*∆ mutant. CFU counts were determined from cultures of tissue homogenates of five animals per group. (**C**) MPO content in kidney of immunosuppressed ICR mice infected with *C. glabrata* WT or *icl1*∆ mutant. MPO content were determined from cultures of kidney homogenates of five animals per group. *p < 0.05 was considered statistically significant relative to WT strain.

To investigate the relevance of *ICL1* to the virulence of *C. glabrata in vivo*, the *icl1*∆ mutants were tested in a mouse model of invasive candidiasis. In this survival assay, equivalent doses of *C. glabrata* WT and *icl1*∆ (2 × 10^8^ cells) were administered to immunocompromised Institute of Cancer Research (ICR) mice via lateral tail vein injection, and the mice were monitored for up to 21 days. Infection with the *C. glabrata* WT strain resulted in 50% mortality within the first three days and achieved 90% mortality at day 21 post-infection. In contrast, infection with the *C. glabrata icl1*∆ cells only resulted in 40% mortality (Fig. 5A), and the remaining mice survived up to 21 days post-infection. Mantel-Cox log rank analysis of survival curve demonstrated that the disruption of *ICL1* confers a significant attenuation in the pathogenicity of *C. glabrata* in this murine model of invasive candidiasis (*p* < 0.05).

**Figure 6:**
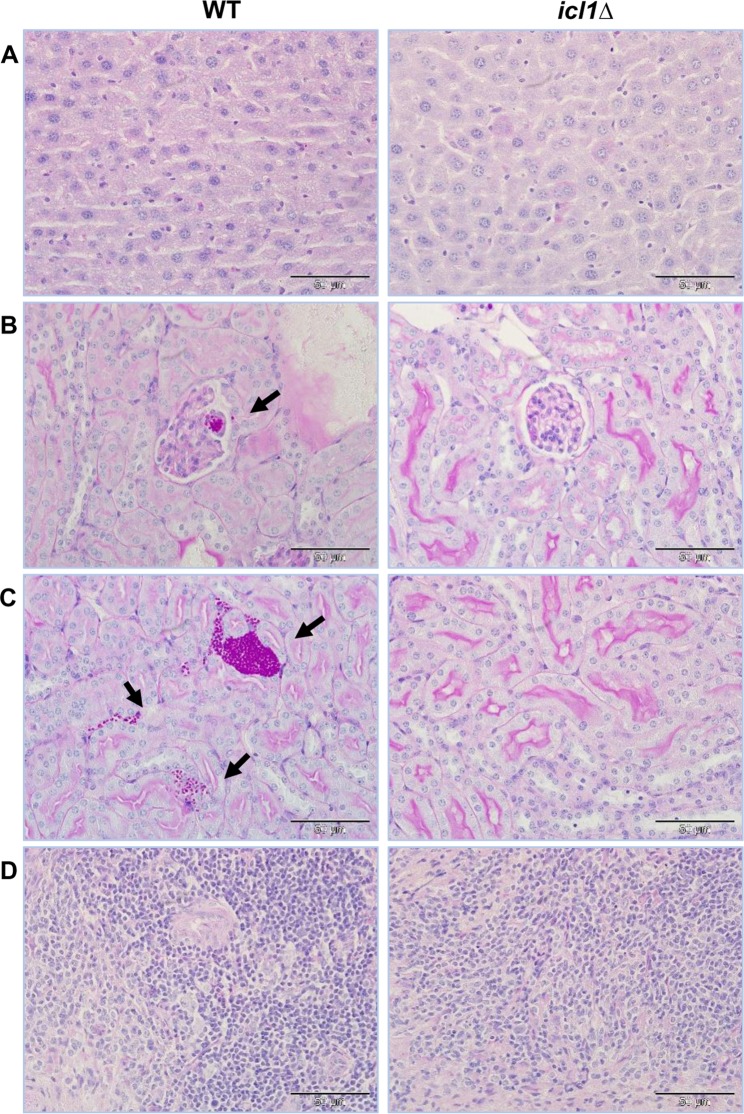
Representative PAS-stained histological sections of different organs from ICR mice. Immunosuppressed ICR mice were infected with 2 × 10^8^ *C. glabrata* WT and *icl1*∆ mutant via lateral tail vein injection, and organs were harvested on day 3 post infection (x400 magnification). (**A**) Liver, (**B**) Renal glomerulus, (**C**) Renal cortex, and (**D**) Spleen. Arrow indicate fungal cells.

Measurements of fungal burden were performed for the *C. glabrata* HTL and *icl1∆* strains from recovered organs at day 3 post-infection. There were no significant differences between the WT and *icl1∆* strains regarding the fungal burdens in the liver (10^6^ CFU/g tissue) and spleen (10^7^ CFU/g tissue). However, the fungal burden in the kidney was greatly reduced in mice infected with the *C. glabrata icl1*∆ mutant, compared to the WT control (*p* < 0.05) (Fig. 5B). Indeed, the kidney fungal burden for the *C. glabrata icl1*∆ mutant was approximately 2 × 10^4^ CFU/g tissue, a significant 63-fold reduction compared to the WT strain. Histopathological sections of kidneys from infected mice showed the presence of *C. glabrata* WT cells in glomeruli on day 3 post infection (Fig. 6). Furthermore, it appeared that *C. glabrata* successfully passed through glomeruli and invaded the renal cortex, as invasion of *C. glabrata* WT cells was observed surrounding renal tubules. In concordance with the results from the survival assay, the disruption of *ICL1* rendered *C. glabrata* less able to invade kidney tissues in these immunosuppressed ICR mice. The MPO content in kidney homogenates was also significantly higher for mice infected with the *C. glabrata* WT strain (1.19 ± 0.035 µg g^−1^) compared to those infected with the *C. glabrata icl1*∆ mutant (0.93 ± 0.056 µg g^−1^) (Fig. 5C).

## Discussion

Nutrient assimilation is essential for the survival and growth of all microorganisms. Hence, for fungal pathogens to thrive in humans, they must adapt effectively to host microenvironments that offer varying nutrients availabilities. Accordingly, fungal pathogens generally display an impressive degree of metabolic flexibility, which contributes to their fitness and pathogenicity *in vivo*. This metabolic flexibility presents a potential target for antifungal drug discovery^29^. In this study, we demonstrate that the glyoxylate cycle, and in particular the enzyme isocitrate lyase, is essential for the metabolic flexibility and pathogenicity of the major pathogen *C. glabrata*. Taking cues from the baker’s yeast *S. cerevisiae*, we anticipated that *ICL1* might be required in *C. glabrata* for the utilisation of fatty acids, ethanol and acetate. Ethanol is believed to enter the cells through passive diffusion, whereas acetate is transported to the cells through the carboxylate transporter, acetate permease^30–32^. Both carbon sources are converted to acetyl-CoA by acetyl-CoA synthetase. Unlike ethanol and acetate, fatty acids like oleic acid are broken down to acetyl-CoA via β-oxidation, which includes the enzymes fatty acyl-CoA oxidase, 3-hydroxyacyl-CoA dehydrogenase and 3-oxoacyl-CoA thiolase^14^. Acetyl-CoA fuels the glyoxylate cycle and gluconeogenesis for glucose production when glucose availability is scarce^14,33^.

We show that disruption of *ICL1* renders *C. glabrata* unable to grow on acetate, ethanol and oleic acid (Figs 1 and 2). These observations were in concordance with the carbon utilisation patterns of *S. cerevisiae*, but they contrast with other *Candida* species like *C. albicans*^28^. Compared to *C. glabrata*, the deletion of *ICL1* in *C. albicans* causes a more significant defect as *C. albicans icl1∆* cells are unable to grow on glycerol as well^28^. Since *C. glabrata* shares a relatively recent common ancestor with many *Saccharomyce*s species^34^, *C. glabrata* might utilise alternative carbon sources in a similar manner to *S. cerevisiae* rather than *Candida* species from the CUG clade. *C. albicans* lies in this CTG clade and this species requires *ICL1* for the utilisation of citrate and glycerol^28^, in addition to fatty acids, acetate and ethanol. However, *ICL* deletion has no effect on the formation of biofilms by *C. albicans* on glucose^35^. Interestingly, in this study, we show that although disruption of *ICL1* has no effects on the biofilm formation of *C. glabrata* on glucose and glycerol, it does significantly impact the biofilm formation of this fungus on acetate, lactate, ethanol and oleic acid (Fig. 3). As demonstrated by the XTT assay, these *C. glabrata icl1∆* cells show reduced metabolic activity when subjected to certain alternative carbon sources. Therefore, reduced biofilm formations observed in acetate, lactate, ethanol and oleic acid-grown *C. glabrata icl1∆* cells are probably attributed to the growth defect of *C. glabrata icl1∆* strains, instead of the impairment of biofilm formation ability. Taken together, this shows that *ICL1* is indispensable for the growth of *C. glabrata* in the presence of some alternative carbon sources.

Macrophages represent a first line of defence during microbial invasion and are responsible for the engulfment and killing of invading pathogens^36^. In this study, we show that the glyoxylate cycle is also crucial to sustain the viability of *C. glabrata* cells trapped within macrophages (Fig. 4). Presumably, *ICL1* disruption prevents *C. glabrata* from reassimilating alternative carbon sources that are generated by fungal autophagy within macrophages, thereby rendering *C. glabrata icl1*∆ cells more susceptible to macrophage killing. This suggests that *ICL1* might be an important virulence factor for *C. glabrata* that is required for the prolonged survival of *C. glabrata* cells following macrophage engulfment *in vivo*. Ramirez and Lorenz (2007) and others^27,28^ have shown that *ICL1* is required for the full virulence of *C. albicans in vivo*. As *C. glabrata* is normally highly resistance to macrophage killing, we postulated that *ICL1* is also crucial for alternative carbon utilisation and survival of *C. glabrata* in the host.

To test this, we investigated the virulence of *C. glabrata icl1*∆ cells using a mouse model of invasive candidiasis. *ICL1* disruption significantly reduced the mortality of infected mice (Fig. 5). Furthermore, fungal burdens in the kidney were significantly reduced, while there was no significant effect of *ICL1* deletion on the fungal burden load in the liver and spleen (Fig. 5). This implies that loss of *ICL1* render *C. glabrata* less competent in kidney invasion. To establish renal candidiasis, *C. glabrata* transits from the renal artery via the afferent arterioles to the glomerulus in renal corpuscle and subsequently infects the renal tubules^37^. Histologic examination of the kidneys of infected mice further confirmed the importance of *ICL1* in kidney invasion of *C. glabrata* (Fig. 6). The reduced virulence of *C. glabrata icl1*∆ cells was further supported by the reduced polymorphonuclear neutrophils (PMN) marker enzyme MPO in the kidney of infected mice (Fig. 5). Taken together, the data indicate that deletion of *ICL1* leads to severe attenuation of *C. glabrata* virulence in mouse model of invasive candidiasis.

In conclusion, our data suggest an essential role for *ICL1* in the utilisation of alternative carbon sources by *C. glabrata*. In addition, we suggest an important role for *ICL1* in promoting the growth and prolonged survival of *C. glabrata* following macrophage engulfment. Most importantly, *ICL1* is required for the full virulence of *C. glabrata in vivo*. Our results could pave a way for the development of new candidate treatments that target Icl1 for antifungal drug development. Further investigation of key metabolic enzymes and regulators of alternative carbon metabolic pathways, such as β-oxidation, glyoxylate cycle and gluconeogenesis in *C. glabrata* are warranted.

## Materials and Methods

### Strains and growth conditions

All *C. glabrata* strains used in this study are listed in Table 1. The triple-auxotrophic strain *C. glabrata* HTL (wild type, WT) was constructed from the reference strain *C. glabrata* ATCC 2001 through the removal of coding sequences of *HIS3*, *LEU2* and *TRP1* using a recyclable, dominant nourseothricin resistance marker SAT1^38^. For construction of three independent strains of *C. glabrata icl1*∆, fusion PCR technique was used to generate gene deletion cassette containing approximately 500 bp of homologous flanking regions for *ICL1*, combined with nourseothricin marker gene, *NAT1*, barcodes identifiers and constant overlap sequences as detailed previously^39,40^. The *C. glabrata* HTL strain was transformed with *ICL1* gene deletion cassette using a modified electroporation method. Nourseothricin-resistant transformants were confirmed for correct deletion of *ICL1* deletion by PCR. Three independently constructed *C. glabrata icl1*∆ were used in this study (Table 1).

**Table 1:** *Candida glabrata* strains used in this study.

*C. glabrata* strains	Genotype	Reference
ATCC 2001	Reference strain	American Type Culture Collection (ATCC)
HTL	Derived from ATCC 2001 *his*::FRT, *leu2*::FRT, *trp1*::FRT	Jacobsen *et al*., 2010
*icl1*∆_a *icl1*∆_b *icl1*∆_c	Derived from HTL *icl1*::NAT1	Schwarzmüller *et al*., 2014

Standard culture media were used, including YPD (Becton, Dickinson and Company, USA): yeast extract (1%, w/v), peptone (2%, w/v), glucose (2%, w/v), agar (1.5%, w/v) and YNB without amino acids (Becton, Dickinson and Company, USA): yeast nitrogen base (0.67%, w/v), ammonium sulfate (0.5%, w/v). Synthetic complete (SC) media were prepared with YNB without amino acids, supplemented with complete supplement mixture (0.2%, w/v) (Formedium, UK), glucose (2%, w/v) and agar (2%, w/v). In growth phenotype assays, glucose was replaced with other alternative carbon sources.

### Growth phenotypes

Growth phenotypes of *C. glabrata* ATCC 2001, WT and *icl1*∆ strains in glucose and alternative carbon sources were investigated on SC media containing glucose (2%, w/v), sodium acetate (2%, w/v), sodium lactate (2%, v/v), ethanol (2%, v/v), glycerol (2%, v/v) or oleic acid (0.2%, w/v) (Sigma-Aldrich, USA) as the sole carbon source. A lower concentration of carbon source was used for oleic acid (0.2%, w/v) as previously described^41^. SC media were incubated at 37 °C for 24 to 96 h (Ramírez & Lorenz, 2007).

For spot dilution assays, *C. glabrata* strains were grown in YPD for overnight at 37 °C, harvested and washed twice with phosphate buffered saline (PBS), pH 7.4 before resuspended into fresh SC media (OD_600nm_ of 1.0) with glucose, acetate, lactate, ethanol, glycerol and oleic acid. Subsequently, cell suspensions were transferred into a sterile 96-well plate and serially diluted five-fold. These dilutions were spotted on SC media supplemented with different carbon sources and incubated at 37 °C for 24 to 96 h.

For microplate-based growth assay, *C. glabrata* strains were grown in YPD for overnight at 37 °C, harvested and washed twice with PBS, pH 7.4 before resuspended into fresh SC media (OD_600nm_ of 0.1) with glucose, acetate, lactate, ethanol, glycerol and oleic acid as sole carbon source. A volume of 200 µl of cell suspension was transferred into a sterile 96-well plate. Growth of *C. glabrata* strains was monitored for 96 h by measuring OD_600nm_ with microtiter plate reader (Dynex Technologies, USA).

### Biofilm formation

Biofilm formation of *C. glabrata* WT and *icl1*∆ mutant in different alternative carbon sources were assessed by using a modified procedure previously described^42^. Briefly, overnight cultures of *C. glabrata* WT and *icl1*∆ mutants were harvested and washed twice with PBS, pH 7.4 before resuspended into fresh SC media (OD_600nm_ of 0.1) with glucose, acetate, lactate, ethanol, glycerol and oleic acid as sole carbon source. A volume of 100 µl cell suspension was dispensed into a pre-sterilized, clear and flat bottomed 96-well polystyrene cell culture plate with low-evaporation lids (Becton, Dickinson and Company, USA). The 96-well plate was covered with its original lid, sealed with parafilm and incubated for 48 h at 37 °C for biofilm formation.

The 96-well plate was washed twice with PBS, pH 7.4 and residual PBS was removed with blotting paper. Biofilm formation of *C. glabrata* strains was quantified by 2,3-bis-(2-methoxy-4-nitro-5-sulfophenyl)-2H-tetrazolium-5-carboxanilide (XTT) reduction assay. A volume of 100 µL solution mixture of 0.5 g/L XTT (Sigma-Aldrich, USA) and 10 mM menadione (10000: 1, v/v) (Sigma-Aldrich, USA) was added to the biofilms. The plate was covered in aluminium foil and incubated in the dark at 37 °C for 3 h. Subsequently, 80 µl of the solution was transferred to a new 96-well plate and OD_490nm_ was measured by using a microtiter plate reader.

### Fungal killing by macrophages

RAW264.7 murine macrophages were cultured in Dulbecco’s Modified Eagle’s Medium (DMEM; Thermo Fisher Scientific, USA) supplemented with 10% (v/v) foetal bovine serum (FBS; Thermo Fisher Scientific, USA) and 1% (v/v) penicillin-streptomycin antibiotics (Nacalai Tesque, Japan) in cell culture flasks (Nunc; Thermo Fisher Scientific, USA) at 37 °C and 5% (v/v) CO_2_. The cells were seeded at a density of 5 × 10^5^ in 12-well tissue culture plates (Becton, Dickinson and Company, USA) for 24 h at 37 °C and 5% (v/v) CO_2_. The cell number was determined by cell counting using a haemocytometer.

For the preparation of *C. glabrata* cells, overnight cultures of *C. glabrata* WT and *icl1*∆ mutant were washed and regrown to mid-exponential phase (OD_600nm_ of 0.5) in fresh YPD. *Candida glabrata* cells were harvested by centrifugation, resuspended in DMEM supplemented with 10% FBS and added to RAW264.7 macrophage at a multiplicity of infection (MOI) of 1: 1 (RAW264.7: *Candida*). Non-phagocytosed *C. glabrata* cells were removed by washing with DMEM after 2 h of co-incubation. Lysates of infected RAW264.7 macrophages were harvested after 2 and 24 h of co-incubation. The cells were lysed with ice-cold sterile deionized water and plated on YPD. Colony-forming-unit (CFU) of intracellular *C. glabrata* cells were counted after incubation at 37 °C for 24 h. Survival ratio of phagocytosed *C. glabrata* cells is defined as (CFU of 24 h Sample/CFU of 2 h Control) ×  100%^13^.

### Mouse model of invasive candidiasis

The virulence of *C. glabrata icl1*∆ mutant *in vivo* was assessed using a modified murine model as previously described^43^. Briefly, female outbred ICR mice (6–8 weeks old, 18–20 g) were obtained from Animal Resource Unit, Faculty of Veterinary Medicine, Universiti Putra Malaysia. The mice were housed in groups of five in individually ventilated cages and offered with standard mouse cubes (Specialty Feeds, Australia) and water *ad libitum*. The mice were first acclimatized under controlled conditions (12/12-h light/dark cycle, 25 °C) for one week before commencement of the studies.

For survival assay, groups of 10 mice were immunosuppressed with cyclophosphamide (200 mg/kg; Merck, Germany) through intraperitoneal injection on day -3 and every fourth day thereafter. Mice were challenged intravenously via lateral tail vein on day 0 with 2 × 10^8^ *C. glabrata* cells in 200 µl of saline 0.9% (w/v). Infected mice were subsequently monitored for sign of infection and humanely euthanized by cervical dislocation under anaesthesia when predetermined end-points were reached (20% body weight loss, laboured breathing, unconscious or moribund state). Survival assay was terminated at day 21 post-infection.

Fungal burdens in tissues were assayed. Groups of 5 mice were immunosuppressed with cyclophosphamide on day -3 and challenged with 2 × 10^8^ *C. glabrata* cells in 200 µl of saline 0.9% (w/v). Infected mice were humanely euthanized at day 3 and organs (liver, spleen and kidney) of each mouse were procured aseptically. The organs were immediately placed in sterile, ice-cold PBS and mechanically homogenized. Subsequently, the serially diluted tissue homogenates were plated on YPD agar. CFU counts were performed after 24 h of incubation at 37 °C. All procedures involving mice were performed in accordance to the protocols approved by the Institutional Animal Care and Use Committee (IACUC), Universiti Putra Malaysia (ethical approval number: UPM/|ACUC/AUPR-034/2017).

### Histology

Harvested organs from infected mice were fixed and kept and in 10% neutral buffered formalin until processed for histology. Fixed organs were paraffin-embedded, sectioned at 5 µm, and stained with periodic acid-Schiff (PAS) according to standard staining protocols. Histological samples were viewed and analysed with an Olympus BX51TRF microscope (Olympus Corporation, Japan).

### Myeloperoxidase quantification

Kidney homogenates of the infected mice were centrifuged twice for 5 min at 4 °C (5000 × *g*) and the supernatants were stored at −80 °C until myeloperoxidase (MPO) quantification. MPO contents were determined by the commercially available mouse MPO enzyme-linked immunosorbent assay (ELISA) kit (Fine Biotech Co., China) according to the manufacturer’s recommendations.

### Statistical analyses

Statistical analyses were performed using GraphPad Prism Version 7.0 Software (GraphPad Software Inc., USA). All experiments were performed at least in three replicates and all data were expressed as mean values from all replicates with the corresponding standard deviations (SD). Differences between control (WT) and sample (mutant) were assessed by unpaired t-test and a *p* < 0.05 was considered to be statistically significant. All significant differences were indicated in the figures, with *^,^ **, and ***indicating *p* < 0.05, <0.01 and <0.001. Comparison and statistical analysis of survival curves was performed using Mantel-Cox log rank test.

## References

[1] Arendrup MC (2010). Epidemiology of invasive candidiasis. Curr Opin Crit Care.

[2] Kullberg BJ, Arendrup MC (2015). Invasive candidiasis. N Engl J Med.

[3] Sobel JD (2016). The emergence of non-*albicans Candida* species as causes of invasive candidiasis and candidemia. Curr Infect Dis Rep.

[4] Beardmore RE (2018). Drug-mediated metabolic tipping between antibiotic resistant states in a mixed-species community. Nat Ecol Evol.

[5] Lamoth F, Lockhart SR, Berkow EL, Calandra T (2018). Changes in the epidemiological landscape of invasive candidiasis. J Antimicrob Chemother.

[6] Wächtler B (2012). *Candida albicans*-epithelial interactions: Dissecting the roles of active penetration, induced endocytosis and host factors on the infection process. PLOS One.

[7] Moyes DL (2016). Candidalysin is a fungal peptide toxin critical for mucosal infection. Nature.

[8] Brunke S, Hube B (2013). Two unlike cousins: *Candida albicans* and *C. glabrata* infection strategies. Cell Microbiol.

[9] Duggan S (2015). Neutrophil activation by *Candida glabrata* but not *Candida albicans* promotes fungal uptake by monocytes. Cell Microbiol.

[10] Seider K (2011). The facultative intracellular pathogen *Candida glabrata* subverts macrophage cytokine production and phagolysosome maturation. J Immunol.

[11] Seider K (2014). Immune evasion, stress resistance, and efficient nutrient acquisition are crucial for intracellular survival of *Candida glabrata* within macrophages. Eukaryot Cell.

[12] Roetzer A, Gratz N, Kovarik P, Schüller C (2010). Autophagy supports *Candida glabrata* survival during phagocytosis. Cell Microbiol.

[13] Ng TS (2015). *SNF3* as high affinity glucose sensor and its function in supporting the viability of *Candida glabrata* under glucose-limited environment. Front Microbiol.

[14] Lorenz MC, Bender JA, Fink GR (2004). Transcriptional response of *Candida albicans* upon internalization by macrophages. Eukaryot Cell.

[15] Kaur R, Ma B, Cormack BP (2007). A family of glycosylphosphatidylinositol-linked aspartyl proteases is required for virulence of *Candida glabrata*. Proc Natl Acad Sci USA.

[16] Ueno K (2011). Intestinal resident yeast *Candida glabrata* requires Cyb2p-mediated lactate assimilation to adapt in mouse intestine. PLOS One.

[17] Cunha DV, Salazar SB, Lopes MM, Mira NP (2017). Mechanistic insights underlying tolerance to acetic acid stress in vaginal *Candida glabrata* clinical isolates. Front Microbiol.

[18] Lorenz MC, Fink GR (2002). Life and death in a macrophage: Role of the glyoxylate cycle in virulence. Eukaryot Cell.

[19] Dunn MF, Ramírez-Trujillo JA, Hernández-Lucas I (2009). Major roles of isocitrate lyase and malate synthase in bacterial and fungal pathogenesis. Microbiology.

[20] Muñoz-Elías EJ, McKinney JD (2005). *Mycobacterium tuberculosis* isocitrate lyases 1 and 2 are jointly required for *in vivo* growth and virulence. Nat Med.

[21] Fang FC, Libby SJ, Castor ME, Fung AM (2005). Isocitrate lyase (AceA) is required for *Salmonella* persistence but not for acute lethal infection in mice. Infect Immun.

[22] Wall DM, Duffy PS, Dupont C, Prescott JF, Meijer WG (2005). Isocitrate lyase activity is required for virulence of the intracellular pathogen *Rhodococcus equi*. Infect Immun.

[23] Lindsey TL, Hagins JM, Sokol PA, Silo-Suh LA (2008). Virulence determinants from a cystic fibrosis isolate of *Pseudomonas aeruginosa* include isocitrate lyase. Microbiology.

[24] Ebel F (2006). Analysis of the regulation, expression, and localisation of the isocitrate lyase from *Aspergillus fumigatus*, a potential target for antifungal drug development. Fungal Genet Biol.

[25] Schobel F (2007). *Aspergillus fumigatus* does not require fatty acid metabolism via isocitrate lyase for development of invasive aspergillosis. Infect Immun.

[26] Rude TH, Toffaletti DL, Cox GM, Perfect JR (2002). Relationship of the glyoxylate pathway to the pathogenesis of *Cryptococcus neoformans*. Infect Immun.

[27] Barelle CJ (2006). Niche-specific regulation of central metabolic pathways in a fungal pathogen. Cell Microbiol.

[28] Ramírez MA, Lorenz MC (2007). Mutations in alternative carbon utilization pathways in *Candida albicans* attenuate virulence and confer pleiotropic phenotypes. Eukaryot Cell.

[29] Ene IV, Brunke S, Brown AJ, Hube B (2014). Metabolism in fungal pathogenesis. Cold Spring Harb Perspect Med.

[30] Paiva S, Devaux F, Barbosa S, Jacq C, Casal M (2004). Ady2p is essential for the acetate permease activity in the yeast *Saccharomyces cerevisiae*. Yeast.

[31] Casal M, Paiva S, Queirós O, Soares-Silva I (2008). Transport of carboxylic acids in yeasts. FEMS Microbiol Rev.

[32] Vieira N (2010). Functional specialization and differential regulation of short-chain carboxylic acid transporters in the pathogen *Candida albicans*. Mol Microbiol.

[33] Turcotte B, Liang XB, Robert F, Soontorngun N (2010). Transcriptional regulation of nonfermentable carbon utilization in budding yeast. FEMS Yeast Res.

[34] Dujon B (2004). Genome evolution in yeasts. Nature.

[35] Ishola OA (2016). The role of isocitrate lyase (ICL1) in the metabolic adaptation of *Candida albicans* biofilms. Jundishapur J Microbiol.

[36] Gilbert AS, Wheeler RT, May RC (2015). Fungal pathogens: survival and replication within macrophages. Cold Spring Harb Perspect Med.

[37] Fisher JF, Kavanagh K, Sobel JD, Kauffman CA, Newman CA (2011). *Candida* urinary tract infection: pathogenesis. Clin Infect Dis.

[38] Reuß O, Vik Å, Kolter R, Morschhäuser J (2004). The *SAT1* flipper, an optimized tool for gene disruption in *Candida albicans*. Gene.

[39] Noble SM, Johnson AD (2005). Strains and strategies for large-scale gene deletion studies of the diploid human fungal pathogen *Candida albicans*. Eukaryot Cell.

[40] Schwarzmüller T (2014). Systematic phenotyping of a large-scale *Candida glabrata* deletion collection reveals novel antifungal tolerance genes. PLOS Pathog.

[41] Piekarska K (2006). Peroxisomal fatty acid beta-oxidation is not essential for virulence of *Candida albicans*. Eukaryot Cell.

[42] Pierce CG (2008). A simple and reproducible 96-well plate-based method for the formation of fungal biofilms and its application to antifungal susceptibility testing. Nat Protoc.

[43] Calcagno AM (2003). *Candida glabrata* STE12 is required for wild-type levels of virulence and nitrogen starvation induced filamentation. Mol Microbiol.

